# A Portable Infrared System for Identification of Particulate Matter

**DOI:** 10.3390/s24072288

**Published:** 2024-04-03

**Authors:** Javier Núñez, Arjen Boersma, Robin Koldeweij, Joseph Trimboli

**Affiliations:** The Netherlands Organisation for Applied Scientific Research, HTC25, 5656 AE Eindhoven, The Netherlands; javier.nunez@tno.nl (J.N.); arjen.boersma@tno.nl (A.B.); robin.koldeweij@tno.nl (R.K.)

**Keywords:** infrared, particulate, crystalline silica, cyclone, waveguide

## Abstract

Occupational exposure to airborne dust is responsible for numerous respiratory and cardiovascular diseases. Because of these hazards, air samples are regularly collected on filters and sent for laboratory analysis to ensure compliance with regulations. Unfortunately, this approach often takes weeks to provide a result, which makes it impossible to identify dust sources or protect workers in real time. To address these challenges, we developed a system that characterizes airborne dust by its spectro-chemical profile. In this device, a micro-cyclone concentrates particles from the air and introduces them into a hollow waveguide where an infrared signature is obtained. An algorithm is then used to quantitate the composition of respirable particles by incorporating the infrared features of the most relevant chemical groups and compensating for Mie scattering. With this approach, the system can successfully differentiate mixtures of inorganic materials associated with construction sites in near-real time. The use of a free-space optic assembly improves the light throughput significantly, which enables detection limits of approximately 10 µg/m^3^ with a 10 minute sampling time. While respirable crystalline silica was the focus of this work, it is hoped that the flexibility of the platform will enable different aerosols to be detected in other occupational settings.

## 1. Introduction

Occupational exposure to particulate matter (PM) is responsible for a wide range of diseases that impact the health of workers and reduce productivity. One of the largest concerns is the threat posed by respirable crystalline silica (RCS), which can cause silicosis, renal failure, lung cancer, pulmonary tuberculosis, and other airway diseases [1,2]. This is a widespread problem as crystalline silica is naturally present in all rock types, usually as quartz, and it accounts for about 12 wt % of the Earth’s crust [3]. The number of industries that use or process silica-containing rocks and minerals is considerable, and it has been estimated that over 2 million workers in the USA and more than 3 million in the European Union have been exposed to silica dust [1,4]. For example construction workers in the USA can encounter this hazard in over 600,000 workplaces, and more than 300,000 people in other industries (e.g., brick manufacturing, mines, foundries, and hydraulic fracturing) have been exposed to crystalline silica dust [2,5].

Hazardous conditions are created when rocks and minerals that contain silica are subjected to high-energy processes like crushing, milling, and grinding. During these activities, respirable particles are released into the air. These particles are of particular concern because they can penetrate deep into the airways and cause inflammation, which can increase the risk of disease with chronic exposure.

There are several common strategies for managing the risks associated with RCS. For example, surfaces can be sprayed with water to minimize dust formation, or ventilation systems can be designed to reduce the PM concentration. Air samples are then collected and analyzed to determine how effective these measures are for controlling the environment. Depending on the results, work procedures might be adjusted, or individuals may be instructed to use face masks or respirators. Unfortunately, this general approach has several disadvantages. For example, industries such as mining and construction include numerous types of activities that occur in different environments, which can significantly affect the local concentration of PM and make proactive management problematic. Instances of overexposure often occur because the RCS concentration is above the level normally anticipated for a particular task. Additionally, sample collection typically involves the use of a filter cassette or impactor, which is operated throughout a work shift and sent to an off-site laboratory for analysis [6]. Because the concentration data are calculated according to the average exposure during approximately 8 hours, specific sources of RCS cannot be identified. Furthermore, there may be extremely high levels of RCS produced by certain processes that are unrecognized because the measurement is averaged across a workday. Another disadvantage is that most lab results are reported weeks after the sample was collected [7]. This extreme delay means that workers cannot adjust their behavior or environment to actively manage their exposure.

There are some commercial devices that provide immediate information about airborne particle concentration. For example, the SidePak Personal Aerosol Monitor (TSI Incorporated, Shoreview, MN, USA) and the PDM3700 Personal Dust Monitor (Thermo Fisher Scientific, Waltham, MA, USA) are designed to be worn on clothing and continuously display data [8,9]. With this information, a user is aware if they are in an environment that exceeds the recommended PM concentration levels. However, the composition of the dust is unknown because commercial devices typically use a light-scattering mechanism (e.g., SidePak) or a gravimetric-based principle (e.g., the PDM3700, which uses a tapered-element oscillating microbalance). The resulting ambiguity makes it difficult to make an accurate hazard assessment, which complicates decisions regarding engineering controls and personal protective equipment. Physical protection against airborne particles provides further complications. Systems involving water spraying, ventilation, and active filtration take significant effort and capital to implement. Masks and respirators are uncomfortable to wear for long periods, especially during strenuous tasks. 

If real-time information about the PM chemical composition were known, more rational safety practices could be implemented and activities/sources of RCS could be identified. Furthermore, bystanders that may be in the general vicinity of PM sources could be better protected, as they may not be aware of the dangers and will not be wearing special equipment.

In our previous study, we described a portable demonstration unit that was designed to obtain the chemical fingerprint of airborne PM using a small FTIR spectrometer [10]. That system, known as the particulate matter chemical identifier (PM-CID), consisted of a miniature cyclone, a hollow waveguide, an air pump, optical fibers, and a compact FTIR unit. Airborne PM was concentrated through the cyclone using the air pump and introduced into the hollow waveguide. The waveguide focused the PM and IR light through the same path, and the material was identified by comparing absorbance peaks to reference measurements. An air-flush system was incorporated into the device to clean the waveguide after every measurement.

In this work, we refine the original concept by adding data processing and hardware improvements to construct an integrated system. This includes compensations for PM refractive index and light scattering, which cause spectra to significantly deviate from traditional attenuated total reflectance (ATR) IR measurements. Additionally, all components were combined in a single housing, and the fiber optical cables were replaced with a free-space optical chamber to reduce optical losses. These modifications make the system both easier to physically deploy in field tests and improve the detection limit because the attenuation associated with the fiber cables is eliminated. Laboratory tests were performed with mixtures of inorganic powders to determine the ability to quantitate crystalline silica in complex environments. The detection limit for crystalline silica was established and compared with the current legal requirements for occupational settings. Field tests were also performed in which the PIM-CID was exposed to aerosols produced in real settings and scenarios, including simulations of a construction site and railway maintenance work.

## 2. Materials and Methods

### 2.1. Materials and Hardware

Aerosols were generated using commercial inorganic powders: amorphous silica (Sipernat 570, Evonik, Essen, Germany), crystalline silica (Merck/MilliporeSigma, Burlington, MA, USA), calcium carbonate (Huber Engineered Materials, Atlanta, GA, USA), and calcium sulphate (Merck/MilliporeSigma, Burlington, MA, USA).

Powders were initially characterized using a laboratory FTIR spectrometer (Thermo Fisher, Nicolet 6700, Waltham, MA, USA) in ATR mode. The prototype CID used a compact TE-cooled FTIR spectrometer having a spectral range of 800–5000 cm^−1^ with a resolution of 4 cm^−1^ (ARCoptix, FTIR FC-4TE, Neuchâtel, Switzerland). 

Particle size and concentration assessments were made using a TSI OPS3330 optical particle counter (TSI Incorporated, Shoreview, MN, USA), suitable for PM between 0.3 and 10 µm.

Aerosols were generated either using a fluidized bed setup [10] or with a custom system employing a 3410U Dust Aerosol Generator (TSI Incorporated, Shoreview, MN, USA) combined with a series of filters, mass flow controllers, and dilution units.

### 2.2. Prototype Design

**Figure 1:**
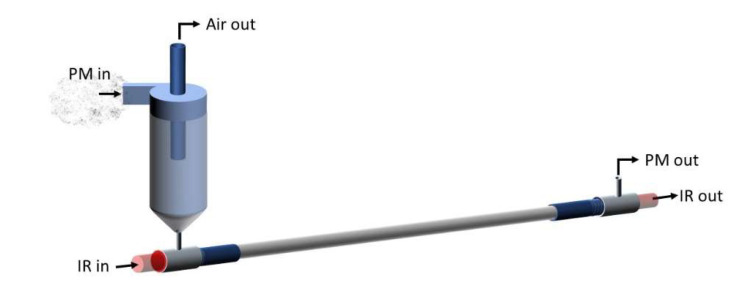
Schematic of the PM-CID main components.

The core technology of the PM-CID has been described in our previous publication [10]. To summarize, this portable system uses a micro-cyclone to concentrate particles into a hollow waveguide for IR detection (Figure 1). Rather than physically capturing particles or using a long-pathlength gas cell (the traditional approaches), the PM-CID design uses the cyclone to concentrate airborne particles into a very small volume of gas. The cyclone efficiency is optimized for a particular application by adjusting the dimensions, the gas flow rate, and sampling time. An Apex 2 vacuum pump (Casella, Bedford, UK) is used to maintain a constant flow through the cyclone for particle separation, and controllers are used to adjust the flow rate. As the particulates are concentrated by the cyclone, they are injected into a hollow waveguide, which directs infrared light and the particulates along the same path. The flow rate in the hollow waveguide is substantially lower than though the cyclone, resulting in a prolonged residence time of the particles in the waveguide. These features enable the component to function like an elongated gas cell while remaining ≤ 10 cm. Using this approach, particulates flowing into the waveguide are sufficiently concentrated to be measured at environmentally and occupationally relevant concentrations (i.e., 10 to 10,000 µg/m^3^) as nearly all PM from the pumped air is concentrated into the waveguide. The waveguide is coupled to a compact FTIR spectrometer to generate mid-infrared absorption spectra, and those results are correlated with particle size/concentration data from a separate, commercial, optical particle counter (AlphaSense, OPC-R1). A MATLAB (v. 2019b) script and an algorithm are then used to identify the particles and assign concentrations.

**Figure 2:**
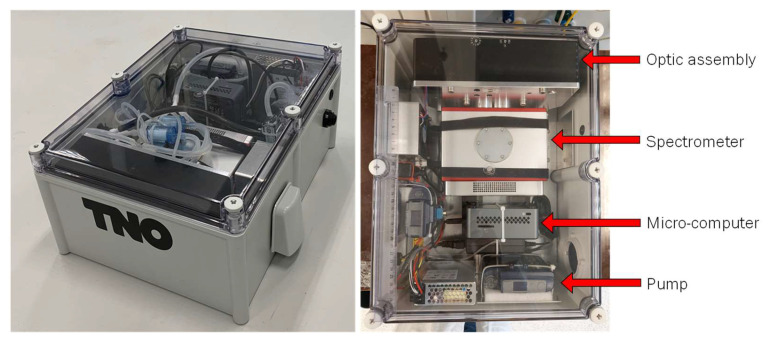
Prototype system (**left**) and top-down image of system (**right**). Tubes required for PM transport and sampling are not present in the top-down image for clarity.

The complete prototype system and a top-down image of the device are shown in Figure 2. An interconnect diagram of the PM-CID is shown in S1. Additional details about the system are provided in the following sections.

#### 2.2.1. Optic Assembly

**Figure 3:**
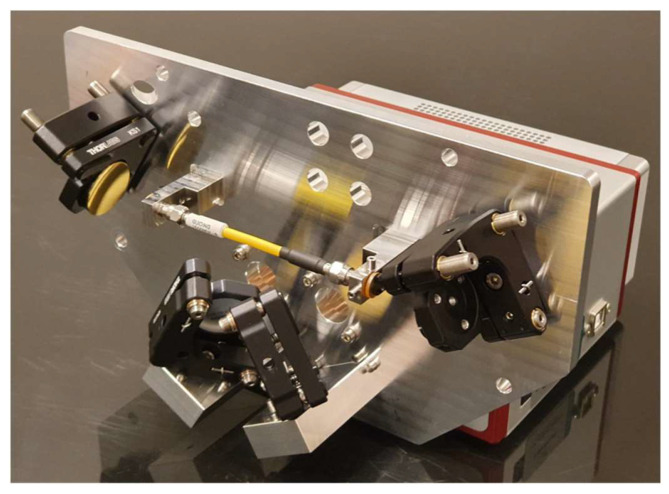
Optic assembly for the PM-CID2 with the cover removed. Two mirrors on the left direct light from the spectrometer through the waveguide, and two mirrors on the right return light to the spectrometer for analysis. IR grade optical windows provide a hermetic seal between the sample volume and the environment.

An optic assembly was designed to focus IR light from the ARCoptix spectrometer into a hollow waveguide using fixed mirrors. This open-path approach increases both the intensity and wavelength range of IR light that is available to interact with particles. Once the light passed through the waveguide, a complementary set of mirrors (Thorlabs, Newton, NJ, USA) returned the IR light to the spectrometer for analysis. The waveguide had a 1.5 mm internal diameter (HFP1500MW-SMA-Yw-0.1m, Guiding Photonics, Torrance, CA, USA) and was equipped with SMA connectors on both ends. All mirrors utilized an unprotected gold coating because a protective layer would have added undesired IR absorbance features to the system. Circular windows transparent to IR light (ZnSe or Ge) were placed on both sides of the waveguide and hermetically sealed with O-rings to prevent leaks. An aluminum baseplate was used to mount all the optical components to make the alignment resistant to thermal fluctuations. A metal cyclone was directly welded to the fiber connector into which PM entered. Figure 3 shows the optic assembly.

Construction of the optic assembly was straightforward, and the component placement was determined by dowel pins that were press-fitted. The kinematic mirror mounts (Thorlabs, Newton, NJ, USA) were adjusted to their nominal positions and assembled with the optical components already mounted. When completed, the optic assembly was mounted on the ARCoptix spectrometer for final integration and alignment. 

#### 2.2.2. Gas Flow

**Figure 4:**
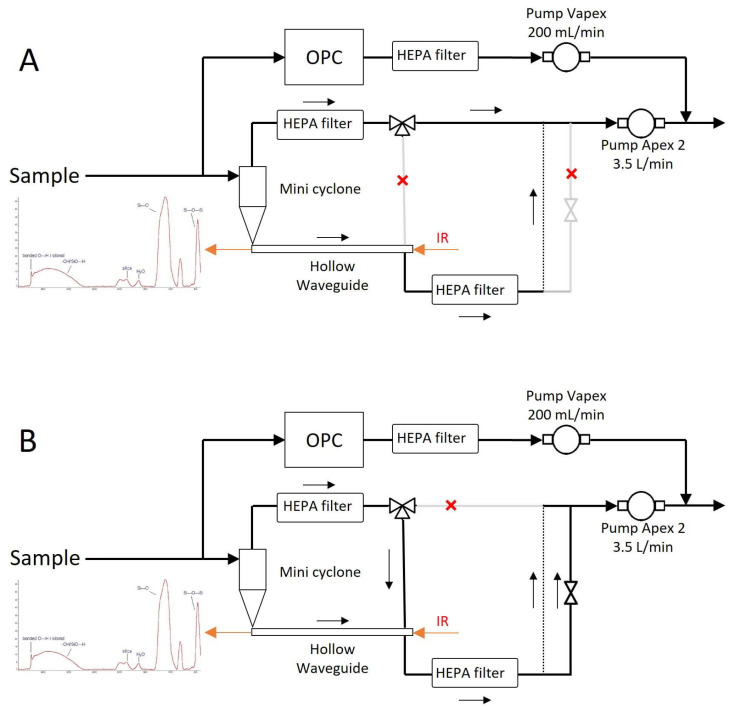
Detailed gas flow diagram for the operational mode (**A**) and the cleaning mode (**B**).

Figure 4 shows the detailed gas flow through the PM-CID. Samples were taken from the environment and split into two paths—one route led to the optic assembly, and the other to an optical particle counter (AlphaSense, OPC-R1). The OPC obtained information about the PM size and concentration, and it was connected to a small pump (Casella, VAPex) operating at 200 mL/min. The OPC was sealed to guarantee air tightness. The flow intended for chemical analysis entered the optic assembly, where it passed through a miniature cyclone and into a hollow waveguide. Two solenoid valves (Parker Hannafin, MX series) were used to control the direction of the flow—particles accumulated in the waveguide during the “operational mode”, and they were flushed with a higher flow in “cleaning mode”. A PCB (Veds Group, Eindhoven, The Netherlands) received commands from a micro-computer to control these valves. A larger pump (Casella, Apex 2) operating at 3.5 L/min drove the flow through this chemical identification compartment.

#### 2.2.3. Cyclone Design and Optimization

To obtain accurate measurements, preferably >95% of the particles above 0.5 µm should be collected. In this section, we describe a redesign of our earlier cyclone that improves the collection efficiency significantly. The design of this cyclone was based on the work of Kenny et al. [11,12] and has been described in our earlier publication [10]. The starting points for the cyclone parameter values from our original study are listed in Table S1.

In the current study, numerical simulations were performed using COMSOL, making use of a 3D finite element method for the Navier–Stokes equations. A v2-f turbulence model was used to capture the anisotropy near the cyclone walls. After obtaining a steady flow field, particles were tracked via a one-way coupled Lagrangian method. The cyclone cylinder diameter (D_c_) was varied between 13.3 mm, 8 mm, and 5 mm. Further specifications are shown in Table 1.

The collection efficiency curves (Figure S2) are predictions according to the empirical model. They show that as D_c_ decreases, there is increased collection efficiency at small particle size. The results from the simulation (orange points) generally follow the empirical models except for geometries with different ratios between the different dimensions. This is because the blue line follows the original cyclone family, and any change in geometry other than a change of scale results in different simulation points, where the empirical constants can no longer be used.

To further improve the cyclone design, comparable simulations were conducted with:

- A cyclone diameter of 8 mm with h, H, Z, and S scaled accordingly
  ○ Variations in insertion length, S, with this 8 mm cyclone;
- A cyclone diameter of 5 mm with h, H, Z, and S scaled accordingly
  ○ Variations in insertion length, S, with this 5 mm cyclone.

Parameters for these models are listed in Tables S2 and S3 and collection efficiency curves are shown in Figures S3 and S4. The data indicate that when other cyclone geometry dimensions were scaled accordingly, the simulation followed the empirical model curve more closely in the small particle size region. As the insertion length S increases, there is an improvement in the collection efficiency of small particles, even down to submicron size particles. This may be due to the lengthened path for particles to escape from the top outlet. Across the geometries examined in this study, cyclone types Dc08-H1-S3 and Dc05-H1-S3 showed the best performance and have a cut-off size down to ~0.6 µm (Figure S5).

The effect of particle density on collection efficiency ranging from 1.5 to 5 g/cm^3^ was studied in cyclone types Dc08-H1-S3 and Dc05-H1-S3. The flow rate was 3 L/min. The results are shown in Figures S6 and S7. These graphs indicate that the particle density had an effect on the small particle region (<1 µm). The collection efficiency was higher for denser particles.

The effect of flow rate on collection efficiency was evaluated in cyclone types Dc08-H1-S3 and Dc05-H1-S3. The flow rate ranged from 1 to 3 L/min. The results are shown in Figures S8 and S9. These graphs indicate that the flow rate had a strong influence on the collection efficiency, especially in the range of 1 to 2 L/min. When the flow rate was 1 L/min, there were hardly any particles collected for either cyclone type.

The pressure drop is also dependent on the cyclone dimensions, and Figure S10 shows the effect of scaling the cyclone on the pressure drop. The maximum backpressure that the pump can keep up at 3.5 L/min is around 100 mbar, which shows that the smaller geometry Dc05-H1-S3 cannot be used at 3.5 L/min with this pump.

Based on the modeling results, we decided to use a Dc08-H1-S3 cyclone in the prototype, operating at approximately 3.5 L/min, since it provided a compromise between particle collection efficiency and a reasonable pressure-drop value that can be reached with small-sized pumps.

**Figure 5:**
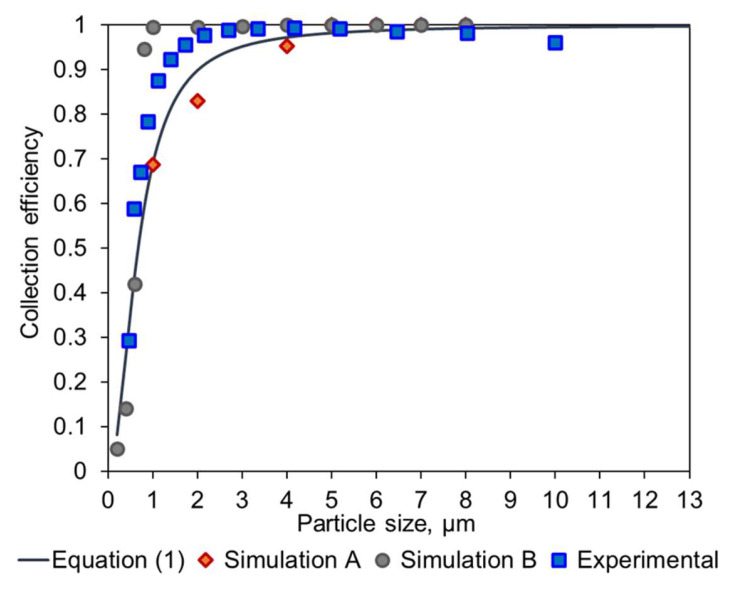
Overlay of the experimental data and the expected collection efficiency values obtained with an empirical model (Equation (1)), the COMSOL modelling of the original Dc08 geometry (Simulation A), and the modified Dc08-H1-S3 geometry (Simulation B).

The resulting prototype was manufactured by means of 3D printing (Shapeways, Eindhoven, The Netherlands), in an acrylate-based polymer, and tested in the lab using a custom setup connected to an OPC Alphasense R1. A comparison of these empirical data and the different methods to calculate the collection efficiency is shown in Figure 5.

Figure 5 shows that the simulation of the original geometry is consistent with the work conducted by Kenny et al. [11,12] and demonstrates the validity of the approach. Furthermore, it can be seen that the modification performed by TNO significantly increased the performance of the cyclone, based on the Simulation B results. Lastly, the experimental data corresponding to this modified geometry show a similar behavior and confirm that the TNO modification provides higher collection efficiency. 

#### 2.2.4. Data Processing

Data were processed using an Intel NUC (Pentium J5005-Quad Core NUC7PJYH2). This micro-computer was used to operate various devices in the PM-CID including the spectrometer, PCBs, wireless dongle (Huawei, Shenzhen, China; model E8372h-320), and OPC. Software native to the spectrometer (ARCspectro Rocket v. 2.4.9.17) and OPC (OPC-R1 Interface software v. 1.0.7713.29019) were used to collect information about particles, and a custom MATLAB script was used to process the data. Commands were issued with an external keyboard, mouse, and monitor, or controlled using TeamViewer run on a cell phone or a remote computer. The wireless dongle enabled users to connect to the PM-CID and view data or change instrument parameters remotely.

**Figure 6:**
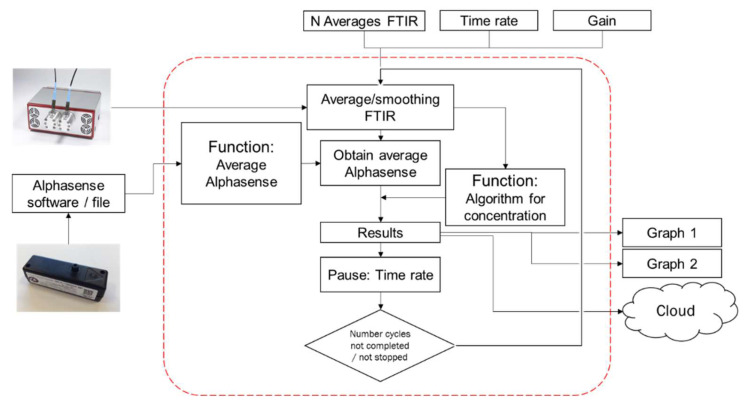
Flow diagram of the data stream in the MATLAB algorithm.

The particle concentration was determined via the OPC and the particle composition via the PM-CID. Both data streams were combined and processed simultaneously by means of an algorithm written in MATLAB. The data flow of this algorithm is shown in Figure 6. The number of scans for the FTIR, the time rate (time resolution), and the detector gain were set by the user to initiate measurements. Next, the system retrieved data from the OPC (at 1 Hz) and the FTIR spectrometer (frequency determined by the sampling rate) simultaneously. The longer the sampling period was, the lower the temporal resolution of the output data and the higher the infrared signal and algorithm accuracy.

## 3. Results

A variety of tests were performed to assess the effects of particle size and concentration, to acquire reference spectra, and to conduct field tests. In this section, we present the most relevant results and findings from the experiments.

### 3.1. Particle Identification

**Figure 7:**
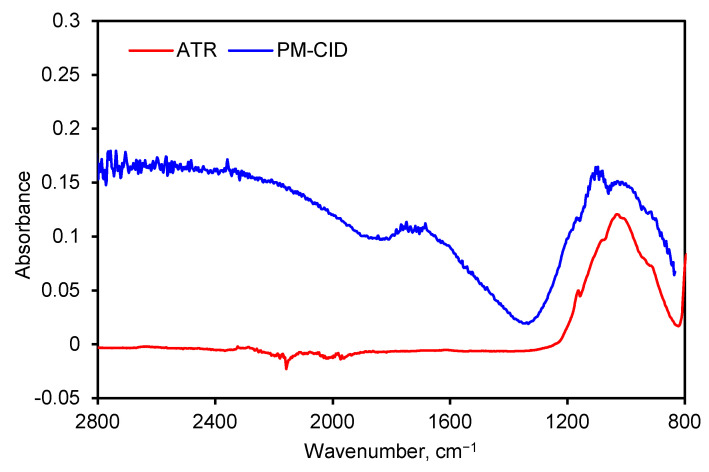
FTIR spectra of ground sandstone using a benchtop spectrometer (ATR) and PM-CID.

Spectra generated by the PM-CID depend on the chemical nature of the dust, their concentration, and the particle size. As a result, data from the PM-CID can differ significantly from a traditional IR measurement. An example of this is shown in Figure 7 where a spectrum of sandstone was taken with an early version of the prototype equipment. Those data were compared with a benchtop ATR spectrometer. The peaks between 900 and 1200 cm^−1^ result from molecular absorption by the sandstone sample. However, the large features in the PM-CID spectrum between 1400 and 2400 cm^−1^ are caused by light scattering, since these do not appear in the ATR spectrum. The minimum at ~1400 cm^−1^ is caused by the Christiansen effect [13,14], which occurs at a wavenumber at which the refractive index of the particles is the same as that of the surrounding medium (in this case air, *n* = 1). This Christiansen feature (CF) is used in geology and aerospace to obtain chemical information from infrared spectra [14,15], and it depends heavily on the silica content of the samples.

Light scattering depends on the size of the particles in comparison with the wavelength of the light. For very small particles, Rayleigh scattering is dominant, but Mie scattering determines the behavior when the particle size is comparable to the wavelength of the light. The OPC showed that typical particle sizes of the dust were between 5 and 10 µm. This is the particle size at which Mie scattering plays a very important role in the transmission and absorbance.

Unfortunately, Mie scattering is a very complex phenomenon, which is difficult to correct for analytically. In the literature, some iterative solutions are proposed which take a very long time to correct even a single spectrum for scattering [16,17,18,19]. A simple description for Mie scattering was proposed by Van de Hulst [20,21,22], and the scattering efficiency of a spherical particle with a constant refractive index was calculated according to:

(1) $$Q_{sca}=2-\frac{4}{p}\sin\left(p\right)+\frac{4}{p^{2}}(1-\cos\left(p\right))$$

in which the parameter *p* includes the particle diameter (*D*), the refractive index (*n*), and the wavelength of the light (λ):

(2) $$p=\frac{2\pi D(n-1)}{\lambda }$$

The scattering cross-section is related to the scattering efficiency by:

(3) $$\sigma_{sca}=N\frac{\pi D^{2}}{4}Q_{sca}=A$$

where *N* is the concentration of particles, and *A* the absorbance.

Unfortunately, we were unable to predict the observed IR spectrum using the Van Hulst model that only incorporates the real part of the refractive index. Bassan solved this problem with a complex iteration protocol, in which the refractive index of the particles was calculated using the Kramers–Kronig approach from the FTIR absorbance [19]. A series of (up to 1000) combinations of refractive index and particle size was used to calculate many possible scattering profiles, and this was fitted to the real data. In an iterative process (that can take many hours), the actual scattering is calculated and used to correct the FTIR data. This is a complex protocol that is not suitable for an in-line sensor, in which we only have seconds to calculate and model the FTIR spectra.

The full theoretical Mie scattering of spheres can be calculated using an open-source calculator (i.e., Mieplot) from the internet [23]. When using a complex refractive index from particles, the Mieplot algorithm calculates the scattering and absorption cross-section (Sext = Ssca + Sabs), which we can compare with the measured data. This is shown in Figure S11 for sandstone particles. Further details about the full Mieplot calculation are provided in the Supplementary Materials.

The last step to interpret the data was calibrating the FTIR responses with pure dust samples. In order to obtain a suitable calibration matrix, several dust samples were tested in the CID. We selected thirteen different samples:

- Five actual stones, whose composition was found on the internet [24,25,26];
- Four pure materials that were assumed to contain almost only a single material;
- Four mixtures that were prepared from these four raw materials.

All samples including their compositions are listed in Table 2. The last four mixtures were prepared from the pure components in a known mixing mass ratio. However, since the particle sizes may be different, the FTIR signal may have deviated from samples that were more homogeneously mixed. Therefore, the actual composition that was used in the calibration may have deviated slightly from the set-point compositions.

FTIR spectra were taken for all the samples listed in Table 2. Most samples were converted into airborne dust and measured using a cyclone and hollow waveguide setup. The stone samples were ground before measuring using a multifunctional tool (Dremel), and the rate of dust production could be controlled by adjusting the rotation speed and pressure of the grinding wheel. Before the spectra were used for the calibration, the contributions of the scattering and water were removed. The resulting spectra are shown in Figure S12.

A calibration matrix was constructed by combining the composition matrix with the FTIR spectra matrix in the wavenumber range between 834–1800 cm^−1^. With this approach, the calibration matrix could convert the FTIR spectra of unknown dust samples to values for the whole composition, including the fraction of crystalline silica. The result of this calculation was a list of the composition:

(4) $$\left[\begin{array}{c} Cr-Silica\\ Am-Silica\\ Carbonate\\ Sulfate\\ Silicates\\ Rest \end{array}\right]=\left[\begin{array}{ccc} {Cr}_{834} & \cdots & {Cr}_{1800}\\ {Am}_{834} & \cdots & {Am}_{1800}\\ {Ca}_{834} & \cdots & {Ca}_{1800}\\ {Su}_{834} & \cdots & {Su}_{1800}\\ {Si}_{834} & \cdots & {Si}_{1800}\\ {Re}_{834} & \cdots & {Re}_{1800} \end{array}\right]\left[\begin{array}{c} {ABS}_{834}\\ \vdots \\ {ABS}_{1800} \end{array}\right]$$

The first matrix to be calculated was the composition matrix, the second matrix was the calibration matrix, in which the wavenumbers between 834 and 1800 cm^−1^ were used, and the third matrix was the FTIR spectrum between 834 and 1800 cm^−1^. We included a “rest” material in the calibration, since not all stones and rocks were composed of only the five materials.

**Figure 8:**
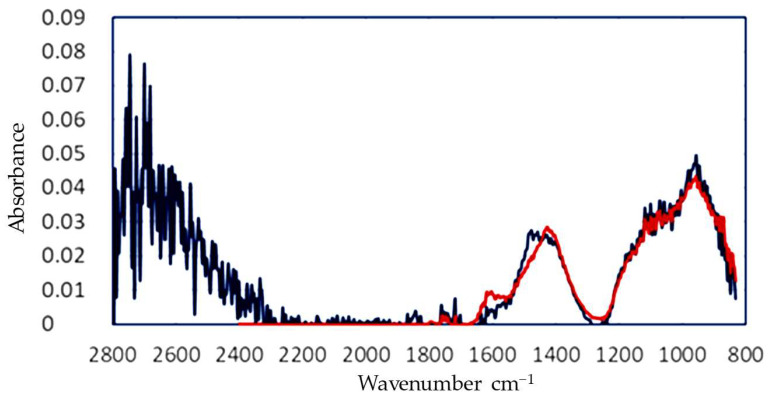
FTIR graph corrected for MIE scattering and water absorption (blue) and fitted to obtain the full composition of the generated dust.

The spectrum that was corrected for scattering and water was then fitted using the calibration matrix obtained from the pure components. In the case of the aerated concrete, the absorption peaks of calcium carbonate at 1400 cm^−1^ and silicate at 950 cm^−1^ were clearly visible. Some of the features were not fitted completely, which may have been caused by unknown components or a non-ideal correction for water. The final results of this calculation are presented in Table 3 and Figure 8.

This approach was applied to other stones and automated, so the PM-CID could identify different types of particles sampled directly from the environment.

Once this calibration process was complete, the integrated system (Figure 2) was exposed in a laboratory to aerosols with a known composition. These tests were used to establish performance characteristics under controlled conditions (i.e., detection limit, response time, recovery time, selectivity via percent composition). A subsequent field trial was conducted in which the PM-CID was compared with traditional measurement techniques to assess its value for real-world applications.

#### 3.1.1. Lab Evaluation

**Figure 9:**
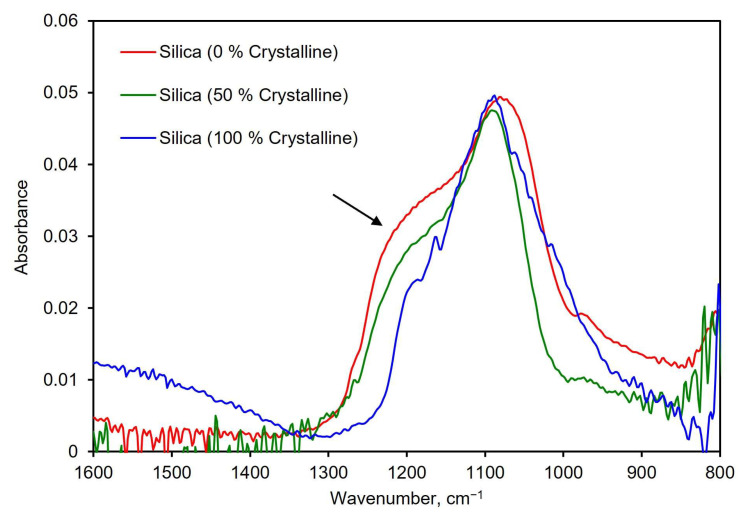
Spectra of three silica samples with different concentrations of crystalline silica. The arrow indicates where the characteristic shoulder in silica changes based on crystallinity.

Differentiating crystalline silica from amorphous silica is essential to assess the risks from exposure to an unknown aerosol. To explore the suitability of the PM-CID for this task, a test was designed in which the PM-CID was exposed to pure silica with different percentages of crystallinity. These powders were made by mixing different mass percentages of amorphous and crystalline silica. Representative spectra from these tests are shown in Figure 9. These results show that the characteristic shoulder in amorphous silica (~1200–1250 cm^−1^) becomes progressively smaller as the amount of crystalline silica increases. While this is a very noticeable difference, it is important to recognize that crystalline silica also has a spectral features between 550–850 cm^−1^ that are particularly suitable for analysis [27,28]. However, the compact spectrometer in our system cannot analyze that region of the mid-infrared spectrum. As a result, we used spectral features at higher wavenumbers to construct our calibration matrix, knowing that positive results are likely to be improved if incorporating these diagnostic peaks.

##### Mixture Characterization for Calibration

The matrix must be calibrated with aerosols of known composition to provide accurate readings of test samples. For this, a dry powder generation system was used to produce specific aerosol compositions for the PM-CID calibration. Trials were performed using the following powders:

- Mix 1—SiO_2_ amorphous/SiO_2_ crystalline (1:3);
- Mix 2—SiO_2_ amorphous/SiO_2_ crystalline/CaCO_3_/CaSO_4_ (1:1:1:1);
- Mix 3—SiO_2_ amorphous/SiO_2_ crystalline/CaCO_3_/CaSO_4_ (3:2:1:4);
- Mix 4—SiO_2_ amorphous/SiO_2_ crystalline (1:1);
- Crystalline silica;
- Amorphous silica;
- Calcium carbonate;
- Calcium sulfate.

Figure S13 shows representative spectra from these tests.

To assess the calibration approach, tests were performed in which the PM-CID behavior was evaluated with real stones, using an older embodiment of our equipment as described in [10]. The sensor was exposed to aerosols released during the cutting of different stones with a Dremel tool, which was equipped with a diamond disk. The spectra obtained with the PM-CID were processed using the previously calibrated algorithm to obtain information about composition, and the results were compared with data obtained from the literature. 

Table 4 shows that the results obtained with the PM-CID follow the trends expected for these two materials. The level of crystalline silica is within 10% of the expected values for both samples, which, combined with the measurement speed (minutes) and portability of the device, provides strong decision support in construction sites in near real time. 

##### Detection Limit of Crystalline Silica

In order to determine the limit of detection for crystalline silica particulates, the PM-CID was exposed to a controlled quartz aerosol in dry air. The most prominent spectral peak of crystalline silica (~1050 cm^−1^) was monitored as a function of concentration. Different exposure durations were used for each test because the infrared signal increases as a function of collection time, due to the adhesion of particles to the inside of the waveguide. Longer durations enable more particles to adhere to the inside of the waveguide, which leads to this behavior.

**Figure 10:**
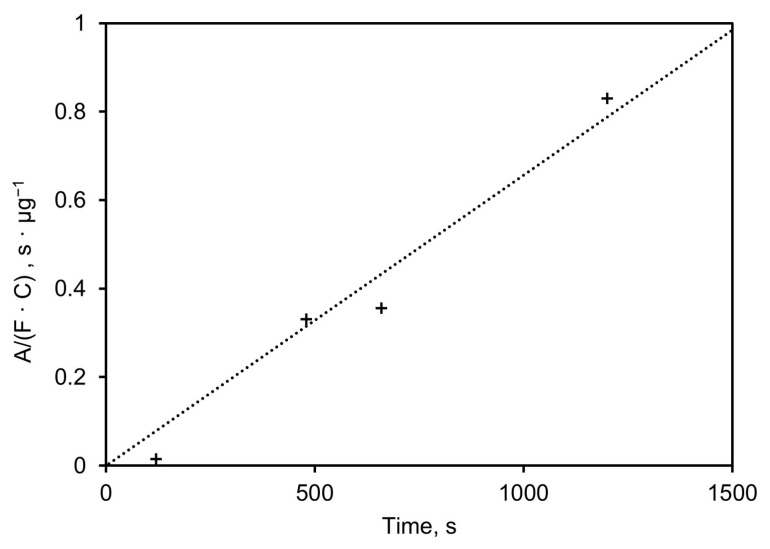
Height of absorbance peak at 1050 cm^−1^ normalized to mass sampling rate plotted vs. sampling time.

Figure 10 shows the height of the absorbance peak at 1050 cm^−1^, normalized to the mass sampling rate (µg/s), plotted versus sampling time in seconds. The slope of the linear regression (*σ*) equals 7.4·10^−4^ µg^−1^, which indicates the absorbance height of the 1050 cm^−1^ (dimensionless) per mass (in µg) of the sampled aerosol. This mass is linearly dependent on the sampling time, the concentration, and the flow rate, as shown in Equation (5).

(5) $$\sigma =\frac{A}{m}=\frac{A}{F\;C\;t}$$

where *σ* is the sensitivity of the system (height at 1050 cm^−1^ per mass in µg), *A* is the absorbance height of the 1050 cm^−1^ feature, m is the mass in µg, *F* is the flow rate in m^3^/s, *C* is the concentration in µg/m^3^, and *t* the time in seconds. 

The limit of detection is dictated by the amount of baseline noise in absorbance, and as the sampling time is increased, the detection limit can be reduced because the absorbance increases for a specific concentration. The noise of the system is, in turn, determined by the amount of light available for the detector (optical efficiency) and the number of scans, which was fixed at 32 for these experiments (20 s). 

**Figure 11:**
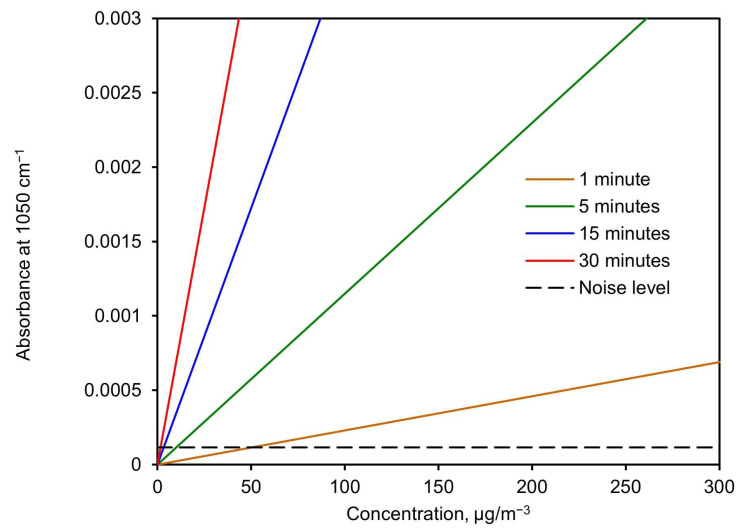
Expected absorbance height at 1050 cm^−1^ vs. concentration for different sampling times.

Figure 11 shows the expected absorbance height at 1050 cm^−1^ versus concentration for different sampling times. This was calculated using the slope value derived from Figure 10 (*σ*) and isolating the absorbance from Equation (5) as a function of the concentration. The noise level (standard deviation) is shown as a discontinuous line to visualize the detection limit. 

**Figure 12:**
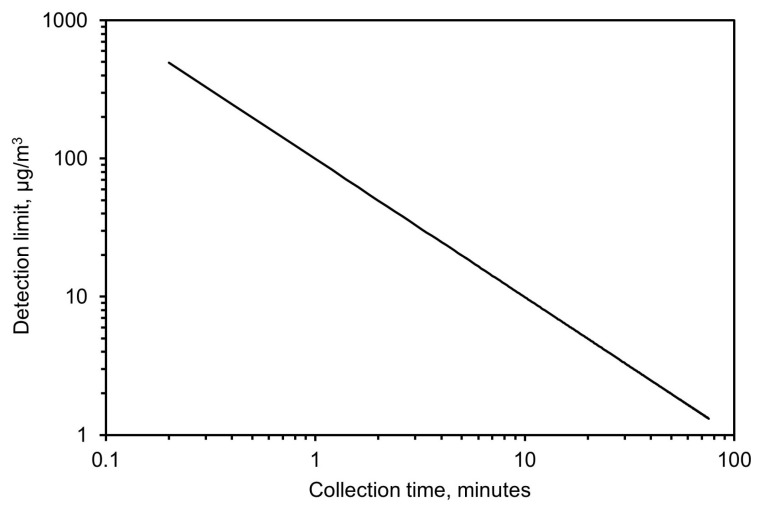
Expected limit of detection based on collection time.

Based on these results, the detection limit was calculated from Equation (5) by calculating the minimum concentration needed, as a function of time, to achieve an absorbance value twice the noise level. This resulted in 99 µg/m^3^ for a 1-min collection time and 10 µg/m^3^ for a 10-min collection time. A complete plot of the detection limit versus collection time for a flow of 3.5 L/min and 32 scans with the spectrometer is shown in Figure 12. 

#### 3.1.2. Field Evaluation—Qualitative and Quantitative Assessments

The PM-CID has been tested in various field locations to validate the functionality of the sensor. One of these tests involved sampling the dust originating from railway stones. The presence of crystalline silica in these stones can pose a threat to workers and the general public as the exposure levels can exceed permissible exposure limits during work activities.

**Figure 13:**
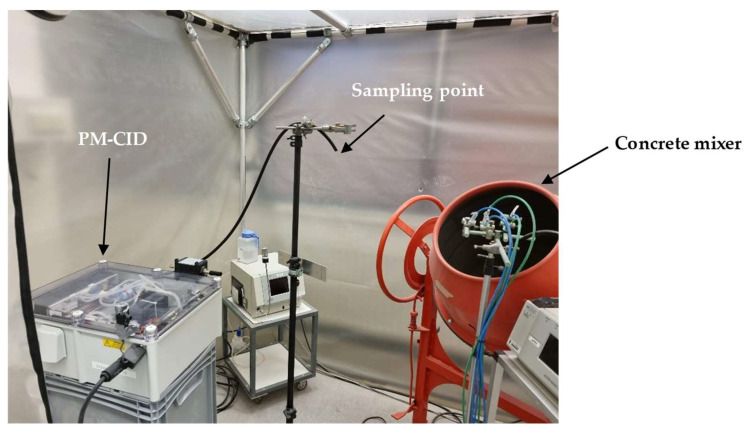
Field test setup for the exposure of the PM-CID to real stone dust from railway stones.

**Figure 14:**
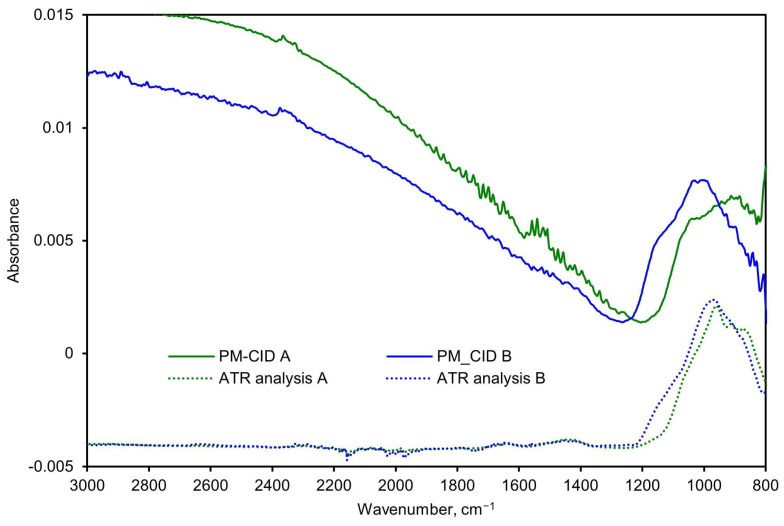
Spectra comparison of two stone samples measured with the PM-CID and a conventional laboratory FTIR spectrometer.

To analyze the potential of the PM-CID for decision support in this setting, a setup was built in which real railway stones were processed in a concrete mixer (Figure 13). The PM-CID was placed a few meters from the mixer and sampled the aerosols originating from the high-energy process. The aerosols were also collected in filters and analyzed in a laboratory FTIR spectrometer. Figure 14 shows the spectra corresponding to two samples originating from two different railway stones, for both the PM-CID (near real-time data) and conventional ATR measurements (post-analysis).

Rather than applying the calibration matrix to calculate the constituents, the spectra were simply inspected qualitatively to determine whether it was possible to quickly detect anomalous features (i.e., as a quality assurance check). As shown in Figure 14, both the PM-CID and the laboratory FTIR measurements show that Sample B produced a more prominent feature at approximately 1150 cm^−1^. Similarly, both measurement techniques show a higher absorbance of Sample A at around 900 cm^−1^. This demonstrates that the PM-CID produces qualitatively similar results as benchtop analytical equipment, with the significant benefit of producing the results in near-real time. As shown in Section 3.1, it is possible to correct for features like scattering (observed in both PM-CID spectra at roughly >1300 cm^−1^) and to further process the stone data to obtain quantitative information about the composition if needed; however, these results show that direct assessments are also possible.

**Figure 15:**
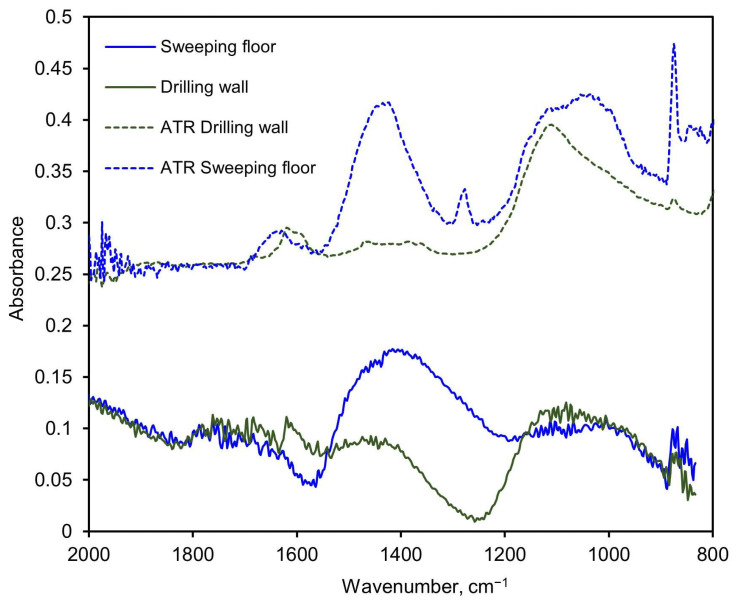
Spectra comparison of two measurements performed with the older embodiment of the PM-CID at an actual construction site, and the corresponding ATR measurements of the aerosols collected in filters. The samples were taken during two different activities: sweeping the floor and drilling the wall.

The legacy PM-CID hardware used for the aerated concrete and sandstone tests was also tested on an actual construction site where a worker performed some routine tasks on walls and the floor. The aerosols were sampled by the PM-CID and simultaneously collected in cassette filters. The materials collected in the filters were analyzed later in the laboratory using ATR, and the concentrations were estimated with a specific matrix calibrated with ATR-only spectra of individual pure compounds. The results obtained with the PM-CID were compared to the concentrations calculated from the ATR spectra of the filter samples, as shown in Figure 15. The floor-sweeping activity produced large, broad IR features around approximately 1100 cm^−1^ and 1400 cm^−1^ in both the ATR and PM-CID measurements. Less prominent features at ~1620 cm^−1^ and 1280 cm^−1^ were observed in the ATR measurement but were absent from the PM-CID. As the ATR samples were generated over a much longer period of time compared with the PM-CID (hours compared with minutes), it is possible that these less prominent features were caused by dust entering the filter cassette later in the measurement, which would be absent from the nearly real-time measurement taken by the PM-CID. In the wall-drilling activity, both systems had a small feature around 1600 cm^−1^ and a larger, broader feature around 1100 cm^−1^. Dips in absorbance were seen in both PM-CID measurements (approximately 1570 cm^−1^ for the sweeping activity and 1240 cm^−1^ for the drilling activity), which may reflect the Christiansen features of those particulate systems.

## 4. Discussion and Conclusions

### 4.1. Cyclone Modifications

Cyclones Dc08-H1-S3 and Dc05-H1-S3 reported in this study are a significant improvement from our prior design. The D_50_ value for the older device was 1.7 µm according to the empirical relation and ca. 2 µm for the numerical model. For environmental and health-related PM measurements, this value is slightly too high—preferably, >95% of the particles above 0.5 µm should be collected. The new cyclones have a D_50_ around 0.6 µm, and the Dc05-H1-S3 design has a particularly high efficiency, with the modeling curve showing ~95% collection efficiency around 0.8 µm. This performance can be further improved by increasing the flow rate beyond 3 L/min, as the trends in Figures S8 and S9 indicate. This adjustment is relatively simple to implement even using inexpensive, personal sampling pumps. These results demonstrate that efficient particle collection (well below PM_2.5_) can be achieved with equipment having small dimensions, low power, and a reasonable cost.

Key design and operating parameters were identified based on the modeling results. It was shown that a small Dc and a long S improved the cyclone collection efficiency for small particles. H needs to be scaled accordingly with Dc. It was also observed that the particle density had an effect on collection efficiency at the small particle region (<1 μm), and that the collection efficiency was higher for denser particles. It was shown that the flow rate had a high influence on the collection efficiency, especially in the range of 1 to 2 L/min. Additionally, it was observed that plastic cyclones tended to accumulate particles during use. This accumulation depended on the type of particles and was probably caused by static charging of the particles when in contact with the plastic surface, in combination with the surface roughness. Another possibility is that the particles became embedded into the internal surface of the cyclone, which can occur when using soft materials such as aluminum or plastic. Once the modelling and stage of the work was completed, a prototype of the most promising design (Dc08-H1-S3) was fabricated in stainless steel. This has several advantages:

- Lower surface roughness;
- No static charging of particles that touch the walls;
- More robust when assembling and using the device.

### 4.2. Prototype Performance

The PM-CID system described in this paper is a significant advancement over our prior design and demonstrates the viability of real-time, infrared particle identification. Based on Figure 11, we see that a particle concentration of 100 µg/m^3^ over 5 min produces roughly a 0.001 absorbance increase. This corresponds to a total sampled mass of 1.75 µg (5 min of 100 µg/m^3^ with flow of 3.5 L/min). 

We believe that two design features are primarily responsible for these performance benefits:

- The optic assembly was designed to focus IR light from the ARCoptix spectrometer into a hollow waveguide using fixed mirrors. This approach increases the amount of IR light (both intensity and wavelength range) that is available to interact with particles.
- The cyclone geometry and material composition significantly improve the PM collection efficiency.

We were able to characterize complex environments with good accuracy using the data processing algorithm. The PM-CID results varied by <10% of the expected values for samples of both sandstone and aerated concrete (Table 4). As the information obtained with the PM-CID was comparable to that found in literature, the sensor was shown to be suitable for near real-time assessments in construction sites when suitable calibration matrices are employed. A simpler approach was taken with separate experiments using railyard stones and construction dusts (Figure 14 and Figure 15). In these cases, we explored whether the PM-CID could be used to find anomalous peaks as a type of quality assessment. During the experiments with railway stones, the general spectral features between the PM-CID and ATR measurements were quite similar—the most notable difference was the rise in the PM-CID baseline due to scattering > 1300 cm^−1^. In the construction dust experiment, there were some small features present in the ATR spectra that were absent from the CID. In this case, it is possible that the much longer sampling time needed for the ATR measurement caused some material to be sampled that was absent during the brief PM-CID test. It is also possible that the breadth of the PM-CID infrared peaks or position of the Christensen feature obscured the smaller peaks.

While these results are promising, there are still some options to improve the classification process in future versions of the PM-CID, particularly when analyzing field samples. One possibility is to use a greater number of each pure compound when constructing the training set. Even small amounts of impurities can have significant effects on the infrared signature of solids [29], and a better understanding of which peaks are consistently present for a particular compound across multiple sources/vendors may aid algorithm development. Additionally, it might be possible to process the data with a different technique. Currently, a calibration matrix is used to convert the FTIR spectra of unknown dust samples into values for the whole composition. Another option is to construct a multivariate regression model to identify the unique features of each component. This approach has been successfully applied to the infrared analysis of minerals in shale rocks, which are quite similar chemically and spectroscopically (e.g., quartz, clay, and feldspars) [30]. The authors of this study also note that it may be possible to minimize the influence from non-calibrated signals (i.e., unanticipated contaminants, organic matter, etc.) by reducing the spectral range to only the most critical regions. Alternatively, it may be possible to use machine learning approaches to train the model with many data sets. Our current approach has been to include a “rest” category in our matrix calculation, but the spectral reduction approach may prove useful. However, it is important to not compromise the predicative capabilities of the system with this approach, as noted by Müller et al. [30]. Additionally, crystalline silica has a spectral features between 550–850 cm^−1^ that are particularly suitable for analysis [27,28]; however, the compact spectrometer in our system cannot analyze that region. By using an alternate spectrometer in the PM-CID or by introducing one or more suitable single-wavelength detectors (e.g., a pyroelectric detector), it would be possible to probe this spectral region and refine the detection algorithm. Finally, it may be possible to reduce spectral features like the contributions from scattering by adjusting the physical way in which particles are deposited into the hollow waveguide. For example, we have been able to generate PM-CID spectra that are more “ATR-like” by causing the particles to accumulate as a sort of wall in front of the fiber optic source before entering the waveguide. In this way, it may be possible to make more robust qualitative assessments or to use calibration matrices that are less dependent on the nature of the dust.

### 4.3. Future Outlook

The current PM-CID is a stand-alone system capable of collecting PM, performing IR analysis, processing spectral data, and sending results to the cloud. The device measures approximately 40 cm × 30 cm × 20 cm, and it can be easily deployed in a variety of field environments. A wearable version of this device has also been developed in which miniaturized components replace the ARCoptix spectrometer. The goal was to achieve sensitivity and selectivity that are complementary with occupational health requirements, while significantly reducing the size (<8000 cm^3^), weight (<5 kg), and cost (<€5k for prototype-scale production).

We have also begun investigating the feasibility of using the core technology for ultrafine particle analysis (<100 nm). These particles are frequently observed in combustion processes as well as from natural sources like biomass; however, real-time characterization, identification, and source apportionment are extremely challenging with current techniques. Also, cyclone separation becomes difficult as Brownian forces tend to dominate aerodynamics at this scale. In this case, it may be more valuable to use an electrostatic technique or other deposition process to achieve the required separation.

## 5. Patents

Several patent applications have been filed based on the combined cyclone, the hollow waveguide, and the infrared detection method.

## Figures and Tables

**Table 1:** Cyclone specifications.

	Dc13 (Original)	Dc08	Dc08-s	Dc05-s	
Dc (mm)	13.3	8	8	5	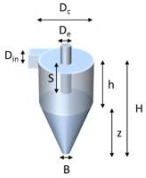
Remarks		Other dimensions keep original scale	De, B are scaled accordingly. H, Z, S, D_in_ keep original scale	De, B are scaled accordingly. H, Z, S, D_in_ keep original scale	

**Table 2:** Stone/PM samples and composition.

	Cr-SiO_2_	Am-SiO_2_	CaCO_3_	CaSO_4_	Ca-Sil	Rest
Sandstone	75%	4%	6%	0	13%	2%
Concrete	60%	10%	10%	0	15%	5%
Quartzite	50%	5%	10%	0	20%	15%
Aerated concrete	30%	10%	20%	5%	20%	15%
Gypsum	0	0	15%	75%	10%	0
Cr-SiO_2_	100%	0	0	0	0	0
Am-SiO_2_	0	95%	0	0	0	5%
CaCO_3_	0	0	100%	0	0	0
CaSO_4_	0	0	0	100%	0	0
MIX1	51%	49%	0	0	0	0
MIX2	30%	22%	0	30%	10%	8%
MIX3	76%	24%	0	0	0	0
MIX4	40%	18%	12%	30%	0	0

**Table 3:** Fitting and calculation results for aerated concrete.

Feature	P1	P2	CF	P3-P7
Diameter	7.6 mm			
Concentration	11 mg/m^3^			
Water		High		
Total silica			64%	
Crystalline silica				39%
Amorphous silica				0%
Calcium carbonate				30%
Calcium sulfate				1%
Calcium silicates				22%
Rest				8%

**Table 4:** Comparison between experimental data using the PM-CID and the literature values for sandstone and aerated concrete samples.

Parameter	Sandstone (exp.)	Sandstone (lit.)	Aerated Concrete (exp.)	Aerated Concrete (lit.)
Diameter	6.6 µm	-	7.6 µm	-
Concentration	27 mg/m^3^	-	11 mg/m^3^	-
Water	High	-	High	-
Total silica	69%	79%	64%	40%
C. silica	67%	75%	39%	30%
A. silica	2%	4%	0%	10%
Ca carbonate	11%	6%	30%	20%
Ca sulfate	3%	0%	1%	5%
Ca silicates	11%	13%	22%	20%
Rest	4%	2%	8%	15%

## Data Availability

The raw data supporting the conclusions of this article will be made available by the authors on request.

## Supplementary Materials

The following supporting information can be downloaded at: https://www.mdpi.com/article/10.3390/s24072288/s1.
