# Supplementary material for: A Portable Infrared System for Identification of Particulate Matter
Source: Sensors (Basel). 2024 Apr 3;24(7):2288. doi: 10.3390/s24072288 (PMC11014306; doi:10.3390/s24072288)
Supplement: Supplementary file 1 [file sensors-24-02288-s001.zip › sensors-2903353-supplementary.pdf]

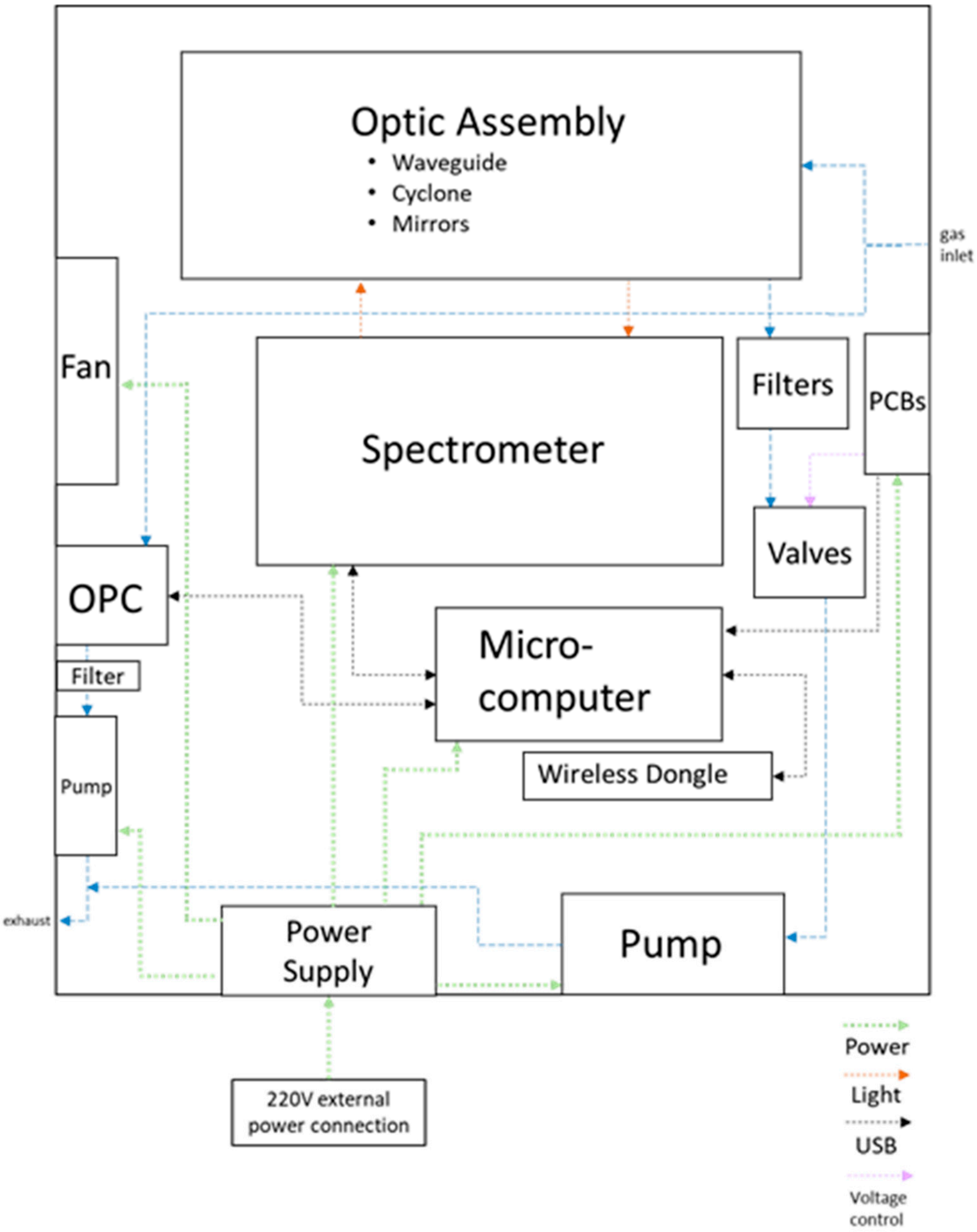

Figure S1: Interface Diagram of the PM-CID with a simplified gas flow path for clarity.

Table S1: The values of the dimensions of the original cyclone

**unit: mm**	**D_c**	**D_e**	**B**	**D_{in}**	**S**	**h**	**Z**	**H**
	13.3	3.3	2.67	1.6	5.3	22.2	26.7	48.9

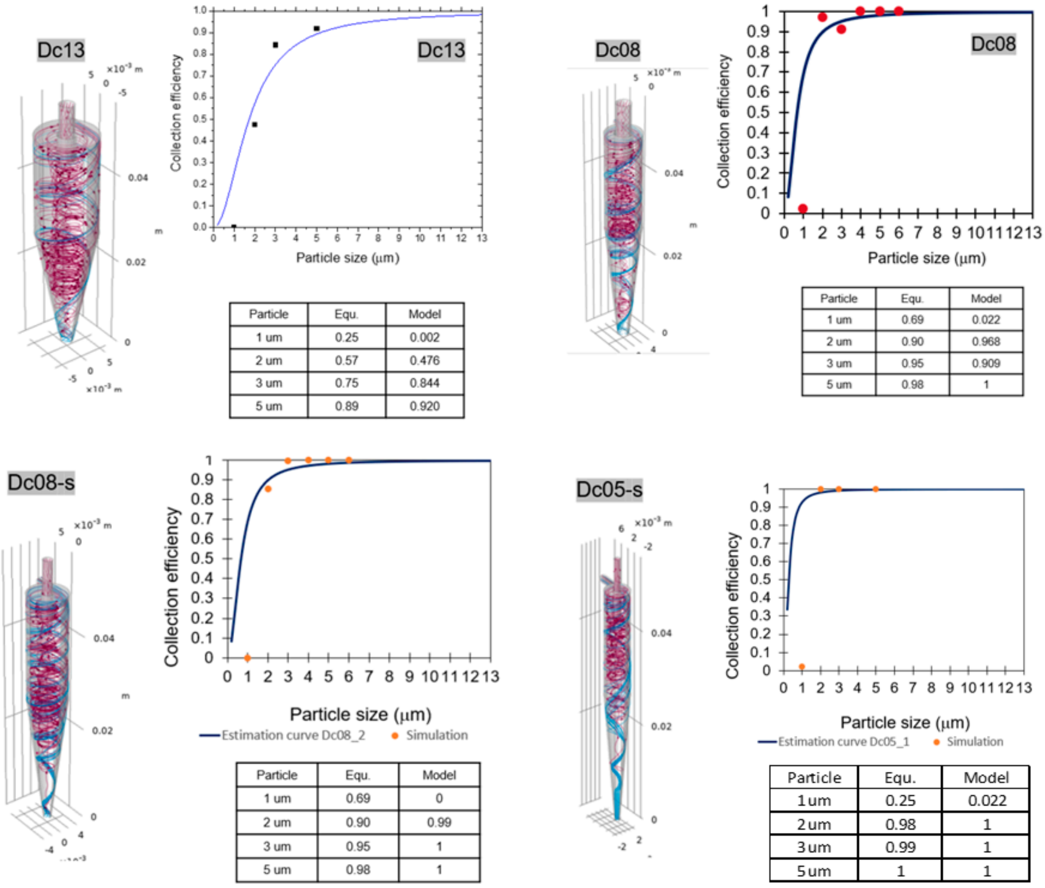

Particle	Equ.	Model
1 um	0.25	0.002
2 um	0.57	0.476
3 um	0.75	0.844
5 um	0.89	0.920

Particle	Equ.	Model
1 um	0.69	0.022
2 um	0.90	0.968
3 um	0.95	0.909
5 um	0.98	1

Particle	Equ.	Model
1 um	0.69	0
2 um	0.90	0.99
3 um	0.95	1
5 um	0.98	1

Particle	Equ.	Model
1um	0.25	0.022
2um	0.98	1
3um	0.99	1
5um	1	1

Figure S2: The airflow and particle collection efficiencies of the Dc13, Dc08, Dc08-s, and Dc05-s cyclones.

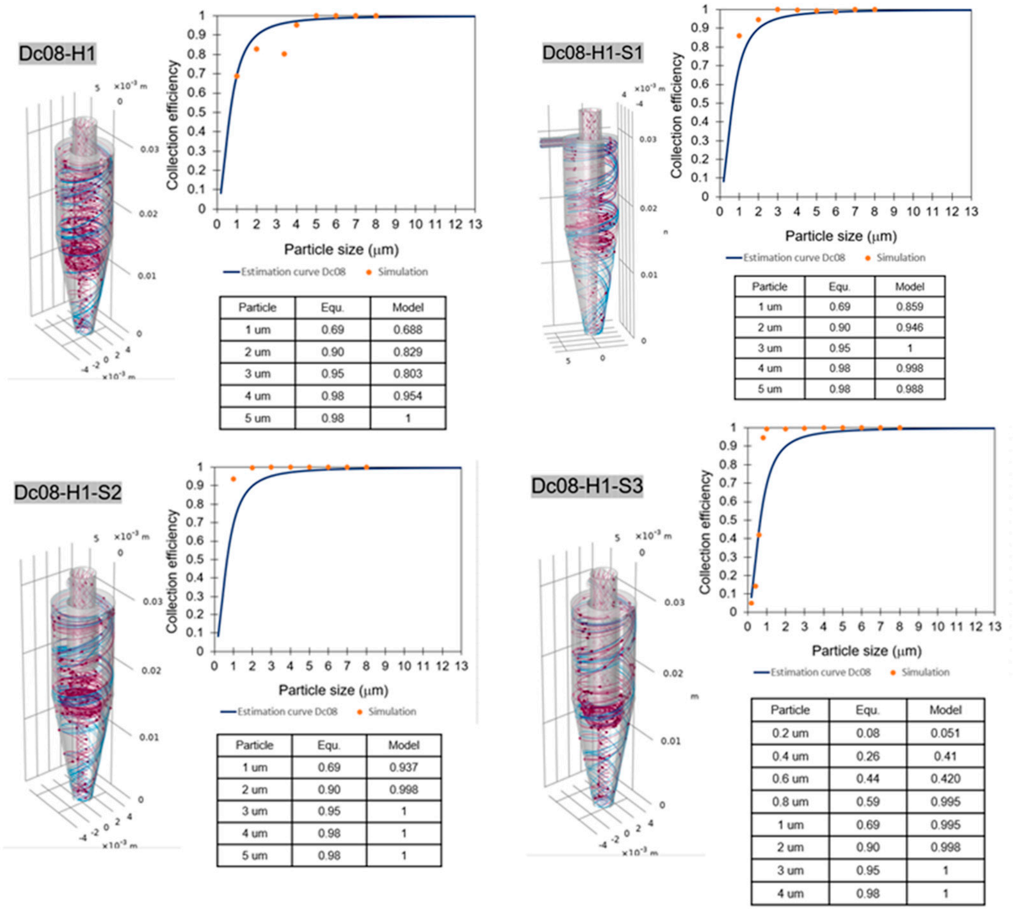

Dc08-H1:

Particle	Equ.	Model
1 um	0.69	0.688
2 um	0.90	0.829
3 um	0.95	0.803
4 um	0.98	0.954
5 um	0.98	1

Dc08-H1-S1:

Particle	Equ.	Model
1 um	0.69	0.859
2 um	0.90	0.946
3 um	0.95	1
4 um	0.98	0.998
5 um	0.98	0.988

Dc08-H1-S2:

Particle	Equ.	Model
1 um	0.69	0.937
2 um	0.90	0.998
3 um	0.95	1
4 um	0.98	1
5 um	0.98	1

Dc08-H1-S3:

Particle	Equ.	Model
0.2 um	0.08	0.051
0.4 um	0.26	0.41
0.6 um	0.44	0.420
0.8 um	0.59	0.995
1 um	0.69	0.995
2 um	0.90	0.998
3 um	0.95	1
4 um	0.98	1

Figure S3: The airflow and particle collection efficiencies of the Dc08-H1, Dc08-H1-S1, Dc08-H1-S2, and Dc08-H1-S3 cyclones.

Table S2: Specifications for Dc08 cyclones.

	**Dc08-H1**	**Dc08-H1-S1**	**Dc08-H1-S2**	**Dc08-H1-S3**
Dc (mm)	8	8	8	8
S	S=0.24h (original)	S=1/2h	S=2/3h	S=h
Remarks	H, h, Z, S scaled accordingly			

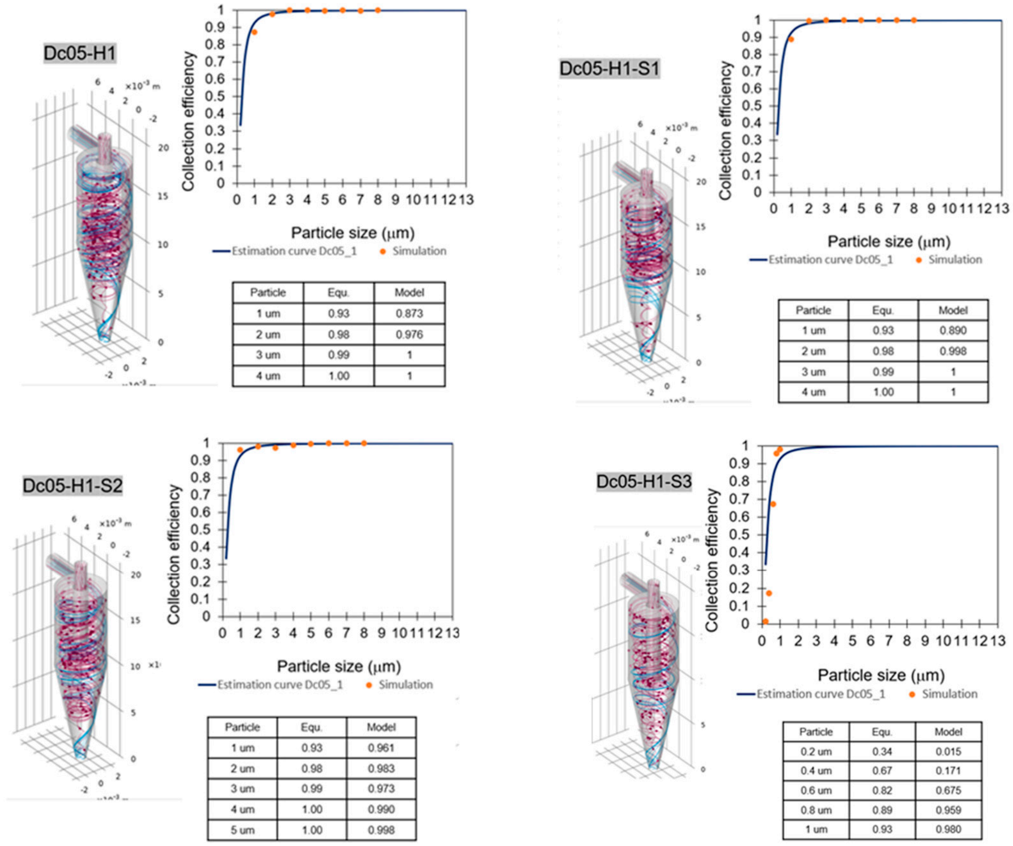

Particle	Equ.	Model
1 um	0.93	0.873
2 um	0.98	0.976
3 um	0.99	1
4 um	1.00	1

Particle	Equ.	Model
1 um	0.93	0.890
2 um	0.98	0.998
3 um	0.99	1
4 um	1.00	1

Particle	Equ.	Model
1 um	0.93	0.961
2 um	0.98	0.983
3 um	0.99	0.973
4 um	1.00	0.990
5 um	1.00	0.998

Particle	Equ.	Model
0.2 um	0.34	0.015
0.4 um	0.67	0.171
0.6 um	0.82	0.675
0.8 um	0.89	0.959
1 um	0.93	0.980

Figure S4: The airflow and particle collection efficiencies of the Dc05-H1, Dc05-H1-S1, Dc08-H1-S2, and Dc08-H1-S3 cyclones.

Table S3: Specifications for Dc05 cyclones.

	**Dc05-H1**	**Dc05-H1-S1**	**Dc05-H1-S2**	**Dc05-H1-S3**
Dc (mm)	5	5	5	5
S	S=0.24h (original)	S=1/2h	S=2/3h	S=h
Remarks	H, h, Z, S scaled accordingly			

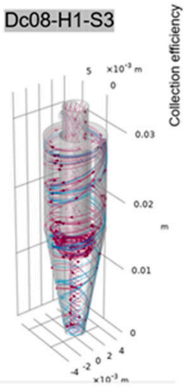

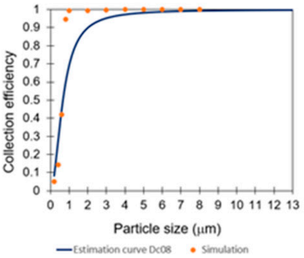

Particle	Equ.	Model
0.2 um	0.08	0.051
0.4 um	0.26	0.41
0.6 um	0.44	0.420
0.8 um	0.59	0.995
1 um	0.69	0.995
2 um	0.90	0.998
3 um	0.95	1
4 um	0.98	1

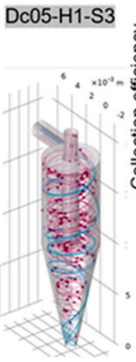

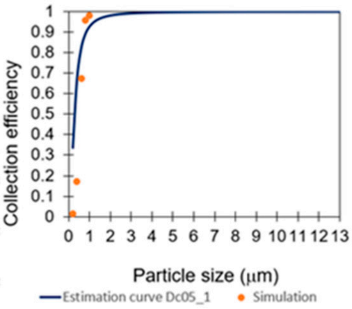

Particle	Equ.	Model
0.2 um	0.34	0.015
0.4 um	0.67	0.171
0.6 um	0.82	0.675
0.8 um	0.89	0.959
1 um	0.93	0.980

Figure S5: The airflow and particle collection efficiencies of the Dc08-H1-S3 and Dc05-H1-S3 cyclones.

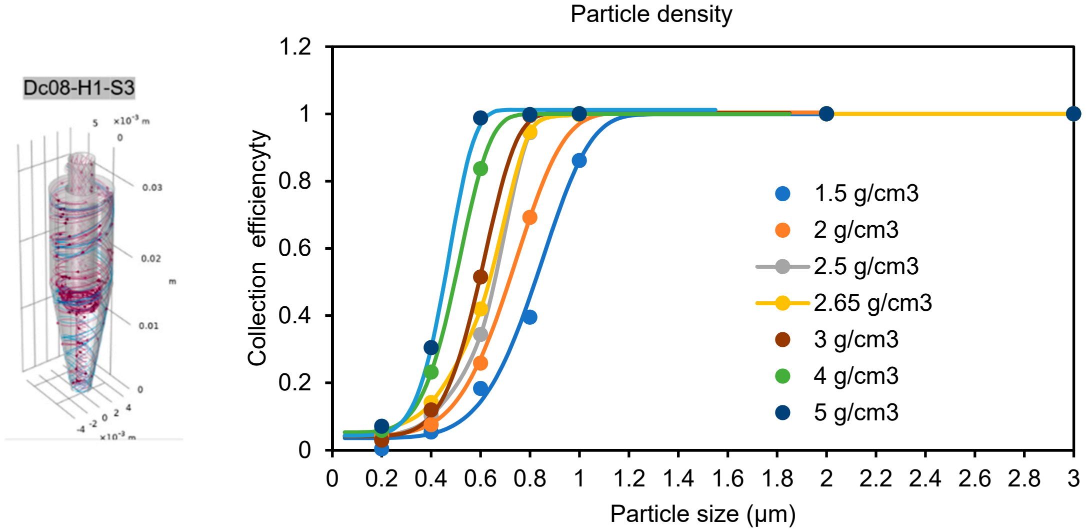

Figure S6: The effect of particle density on collection efficiency for the Dc08-H1-S3 cyclone

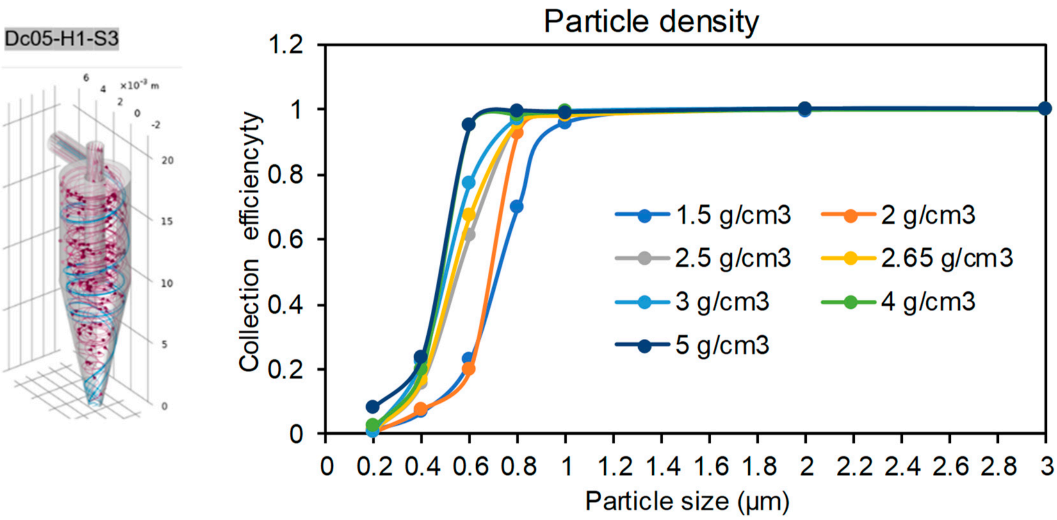

Figure S7: The effect of particle density on collection efficiency for the Dc05-H1-S3 cyclone

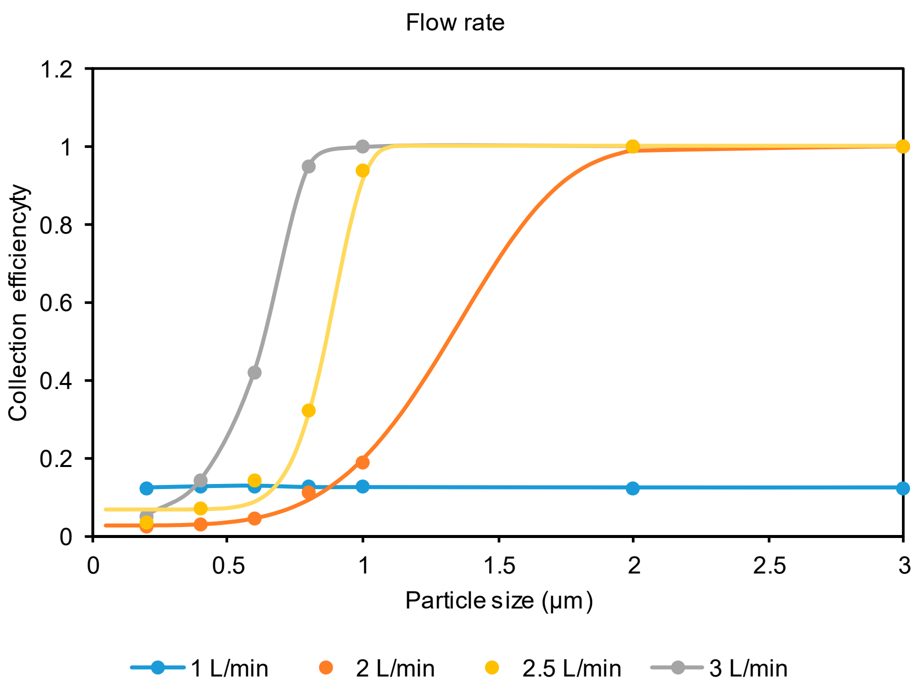

Figure S8: The effect of flow rate on collection efficiency for the Dc08-H1-S3 cyclone.

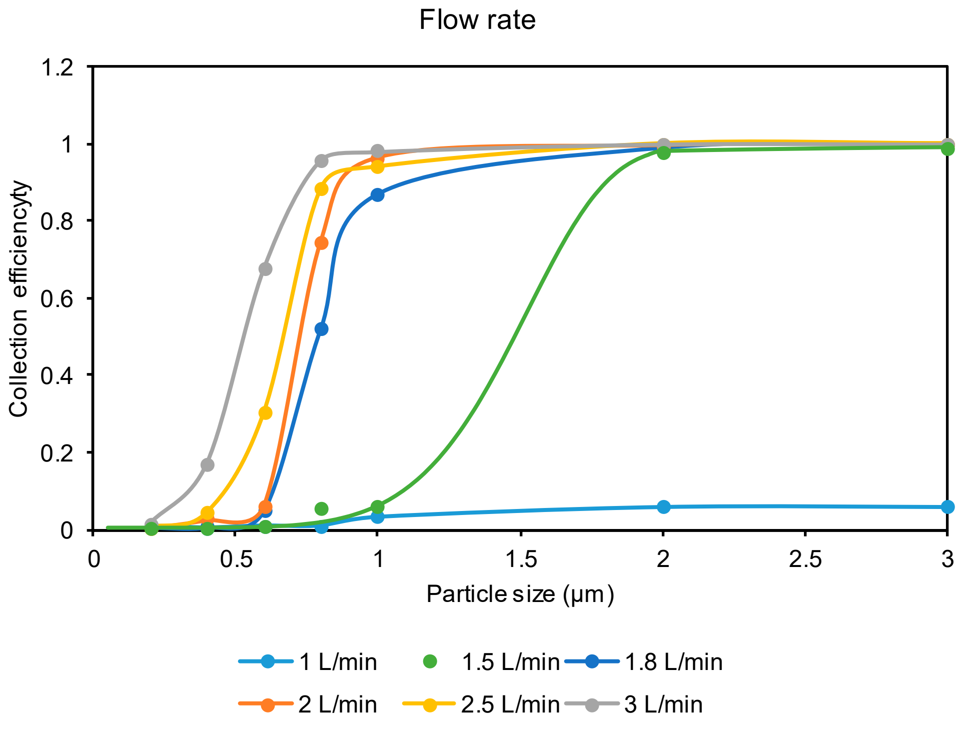

Figure S9: The effect of flow rate on collection efficiency for the Dc05-H1-S3 cyclone.

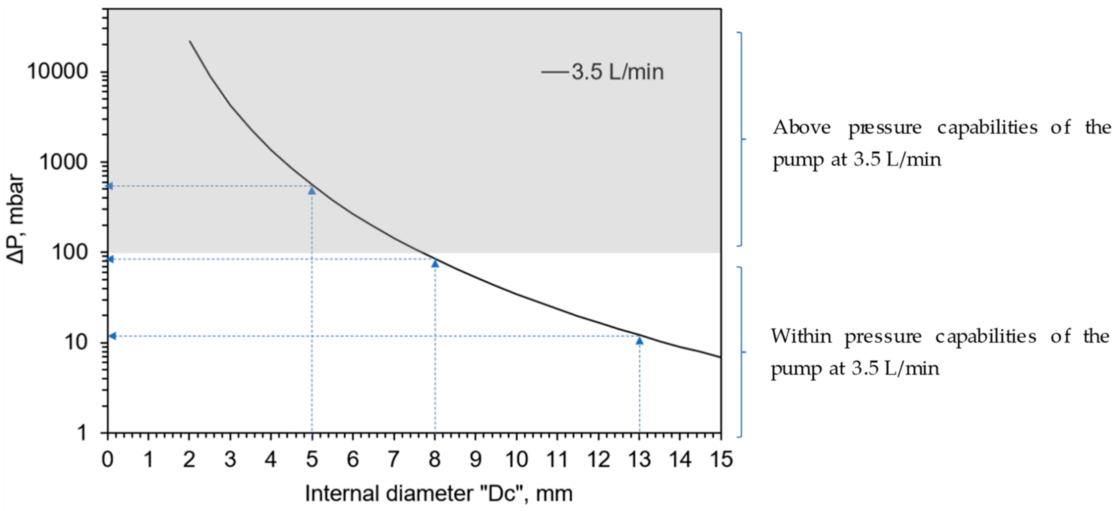

Figure S10: The effect cyclone size on the pressure drop at 3.5 L/min.

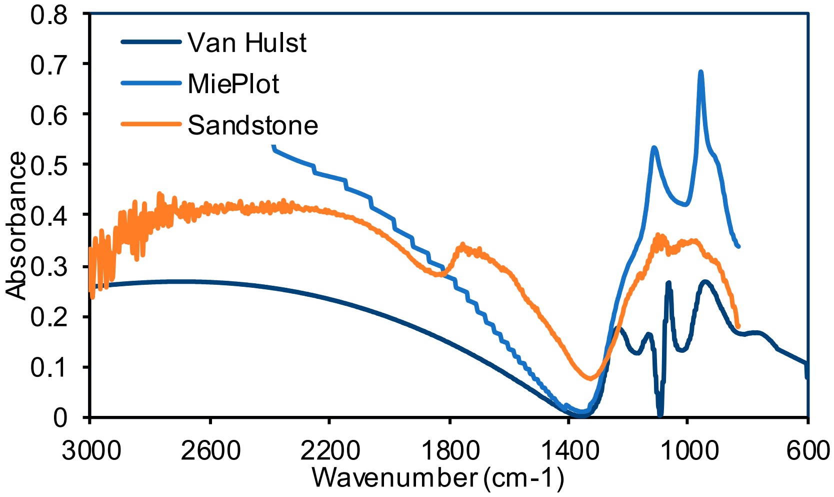

Figure S11: Comparison between Van Hulst, Mieplot, and measured absorbance for 6 µm sandstone particles

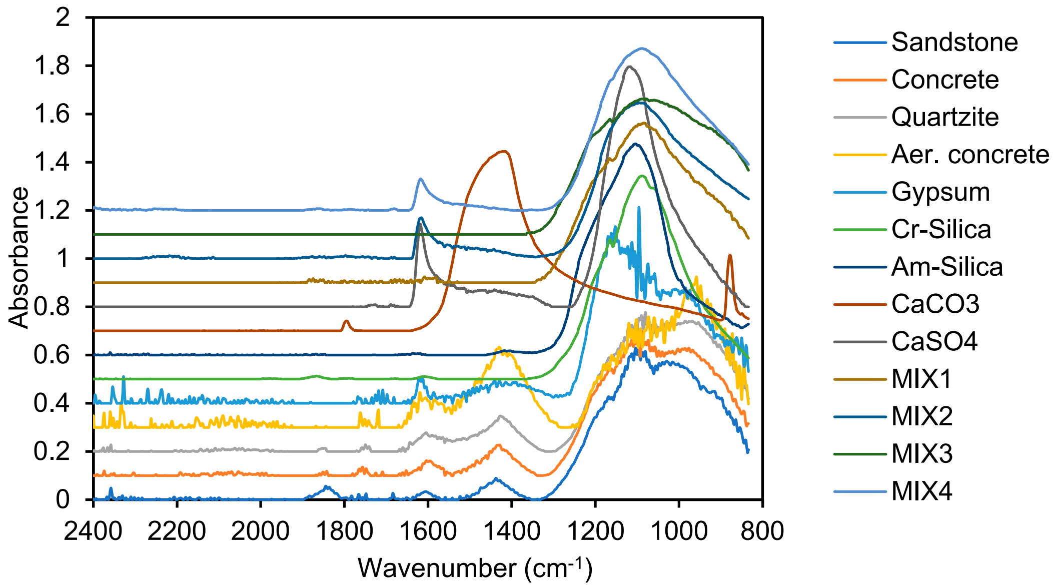

Figure S12: Overview of FTIR spectra from the 13 calibration samples in which the water and scattering contributions were already removed.

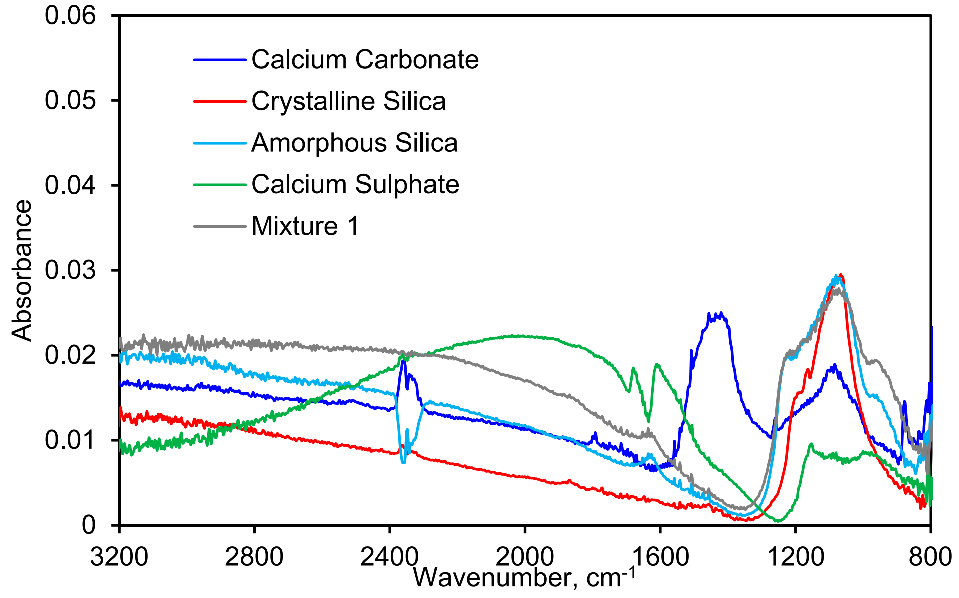

Figure S13: Raw spectra from individual components and Mixture 1.

Calculation Approach with Mieplot

The full Mieplot calculation resembles the actual sandstone measurement better than the simplified van Hulst calculation. In order to derive the data from the measurement, it would be preferable to fit the spectra to the complex Mie scattering model. Since this is virtually impossible, we used an alternative approach:

- First, a series of reference spectra were generated using MiePlot, having varying particles sizes and complex refractive indices from the expected materials.

- Then, several characteristic parameters were derived from the FTIR spectrum, e.g., CF, peak wavelength, slopes, etc.

• These characteristic parameters were used to obtain a first indication of particle size, composition, etc.

• Finally, the reference spectra were fitted to the measured spectra to obtain a more accurate value of the composition.

Various features in the FTIR absorbance spectrum were assigned using this process. Figure S14 shows the data from the sandstone particles along with a fit generated with this approach. Table S4 summarizes the relevant spectral features that were observed with this material.

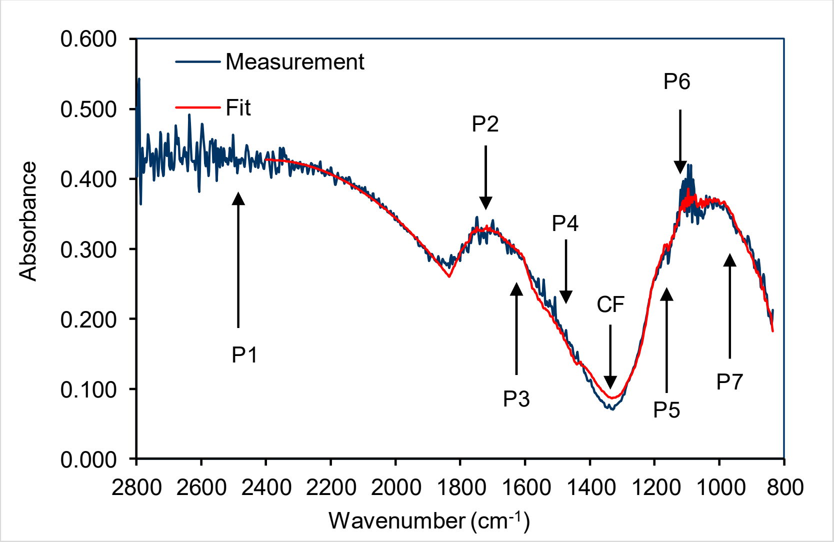

Figure S14: Absorbance spectrum of sandstone with the most relevant features noted.

Table S4: Assignment of relevant spectral features in the FTIR spectrum of sandstone.

Feature	*Origin*	*Information on parameter*
Peak P1	Scattering	Particle size and concentration
Peak P2	Water	Free water content
Peak P3	Water	Crystal water
Peak P4	Absorption	Carbonate (not visible in this plot)
Peak P5	Absorption	Crystalline silica
Peak P6	Absorption	Total silica
Peak P7	Absorption	Phosphate and silicates
CF	Christiansen	Refractive index and silica content

Based on Figure S14, it was clear that the recorded FTIR spectrum can only be fully interpreted when all features are taken into account. This included:

- The Christiansen feature (CF)—the raw data between 1200-1400 cm^{-1} were fitted with a quadratic function to calculate the minimum value of the CF and the corresponding wavenumber. The minimum value was subtracted from the raw data.

- Mie scattering—the scattering feature between 1900–2400 cm^{-1} was fitted, and the peak position and height were used to calculate the particle size and concentration.

- Free water—the free water peak was also fitted with a quadratic function and subtracted from the data. This leaves a spectrum that only contains absorption from the particles in the dust samples.
